# Silane Effect of Universal Adhesive on the Composite–Composite Repair Bond Strength after Different Surface Pretreatments

**DOI:** 10.3390/polym12040950

**Published:** 2020-04-19

**Authors:** Gioia Michelotti, Maria Niedzwiecki, Darius Bidjan, Phoebe Dieckmann, Shengjile Deari, Thomas Attin, Tobias T. Tauböck

**Affiliations:** Clinic of Conservative and Preventive Dentistry, Center for Dental Medicine, University of Zurich, 8032 Zurich, Switzerland; maria.niedzwiecki@uzh.ch (M.N.); darius.bidjan@bluewin.ch (D.B.); phoebe.dieckmann@zzm.uzh.ch (P.D.); Shengjile.Deari@zzm.uzh.ch (S.D.); thomas.attin@zzm.uzh.ch (T.A.); tobias.tauboeck@zzm.uzh.ch (T.T.T.)

**Keywords:** composite repair, surface treatment, universal adhesive, silane, microtensile bond strength

## Abstract

This study investigated the impact of a separate silanization step on the repair bond strength of composite substrates using a universal adhesive after various mechanical surface treatments. Composite specimens were aged and exposed to the following mechanical roughening treatments: diamond bur abrasion, aluminum oxide sandblasting, or silica coating. The specimens were then either left untreated or conditioned with universal adhesive (Scotchbond Universal), or a silane coupling agent was applied before the use of the universal adhesive or a conventional adhesive (Optibond FL). The conditioned surfaces and fresh substrate (positive control group) were covered with repair composite, and microtensile bond strength testing was performed. The significantly highest bond strength was obtained in the positive control group. Repair bond strength of the universal adhesive applied after a separate silanization step was similar to that without prior silanization, independent of the mechanical surface treatment. Moreover, after silica coating, no significant differences in the repair bond strength were detected among the different adhesive treatments. In conclusion, a separate silanization step before surface conditioning with the universal adhesive does not enhance the repair bond strength. On silica-coated composite substrates, repair bond strength values of the universal adhesive were similar to those of the conventional adhesive.

## 1. Introduction

Nowadays, resin-based composite is the most frequently used restorative material worldwide [1]. Despite the immense progress over the last decades concerning physical and mechanical properties [2,3,4,5,6,7], resin-based composite restorations still fail mainly because of fractures or secondary caries [8,9]. The question then arises whether to replace or to repair the partially insufficient restoration. Repair restorations have several advantages over replacements, as they are less time and cost intensive. Furthermore, they preserve sound tooth tissue and decrease the risk of pulp irritation, and therefore represent a minimal-invasive treatment approach to increase the longevity of restorations [10,11,12,13,14,15].

For a successful repair restoration, adequate mechanical and adhesive pretreatment is required. Mechanical roughening of the composite substrate surface for micromechanical retention has been considered crucial in order to obtain adequate composite–composite repair bond strength [16,17]. Indeed, several studies indicated that pretreatment of the aged composite substrate surface with diamond burs [18], sandblasting with aluminum oxide (Al_2_O_3_) [19,20,21], or silica coating [22,23] improves the repair bond strength by increasing the surface area for bonding with the adhesive resin.

Furthermore, silanization of the substrate surface before application of an adhesive has been shown to enhance the bond strength between the substrate and repair composite [24,25,26]. Silanes contain (i) silanol groups, which react with exposed inorganic filler particles of the aged composite substrate and (ii) organofunctional groups, which react and co-polymerize with methacrylate groups of the repair material. The surface coated with silane is more reactive for the repair resin and forms covalent bonds with it. Moreover, silanes increase the wettability of the composite substrate surface, which improves the infiltration of the bonding agent into the surface microretentions [25,27,28].

The use of universal adhesives is becoming increasingly popular among dentists, since they can be applied as self-etch adhesives, with or without previous selective phosphoric acid etching, or etch-and-rinse adhesives, with similar dentin bond strength recorded for the different application modes [29,30]. In order to expand the indication of universal adhesives, some of these adhesives include a silane coupling agent in their formulation to simplify and expedite a technique-sensitive procedure by saving a separate silanization step, which is recommended for adhesive bonding to ceramic and for repair restorations [24,31]. However, previous studies reported that silane incorporated in universal adhesives was not stable in the long-term because of hydrolysis and dehydration condensation promoted by the low pH of the adhesives [27,32]. As a matter of fact, silane-containing universal adhesives reached lower repair bond strength on ceramic surfaces than when the silane coupling agent and the adhesive were applied separately [33]. The effectiveness of the universal adhesive with integrated silane on the repair bond strength to composite substrate surfaces is hardly investigated [33]. Furthermore, information about the influence of different kinds of mechanical surface pretreatments in combination with the use of universal adhesive on the composite–composite repair bond strength is needed.

Thus, the aim of the present in vitro study was to investigate the effect of applying a silane-containing universal adhesive on the repair bond strength of aged composite after different mechanical surface pretreatments. The null hypotheses tested were that (1) there would be no differences in the repair bond strength between a silane-containing universal adhesive used with or without additional separate silanization and (2) that there would be no differences in the repair bond strength between different types of mechanical surface pretreatment.

## 2. Materials and Methods 

### 2.1. Specimen Preparation

**Figure 1:**
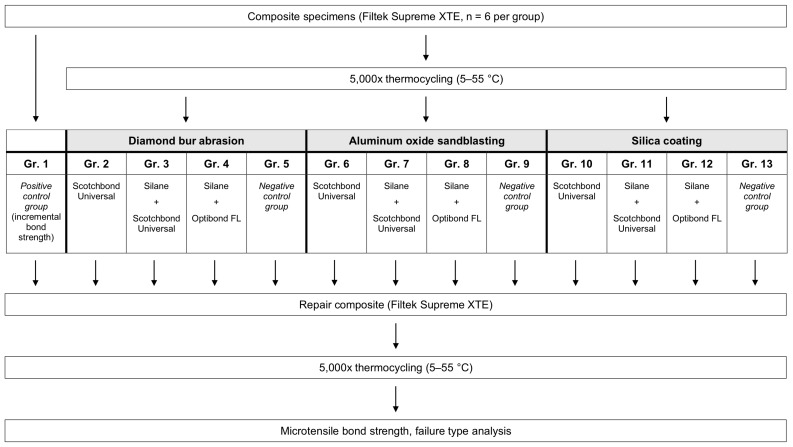
Experimental design of the present study with group denotation for the mechanical and adhesive pretreatments.

The compositions of the main materials used in the present study are given in Table 1. Seventy-eight specimens (diameter: 10 mm) were fabricated by placing three 1.5 mm thick composite increments (Filtek Supreme XTE, 3M, St. Paul, MN, USA; color A4D) on scanning electron microscope (SEM) carriers using cylindrical Teflon molds. To achieve a flat surface, the composite surface was leveled using a PTFE-roller (CompoRoller TM 5300, KerrHawe, Bioggio, Switzerland). Photo-activation was performed for 20 s at 1500 mW/cm^2^ (Elipar Deepcure-S, 3M). Subsequently, the 78 specimens were randomly assigned to 13 groups of six specimens each (Figure 1). Specimens of the positive control group (group 1) were immediately processed further, while all other groups were polished under constant water cooling with 4000-grit silicon carbide (SiC) paper (Buehler-Met II, Buehler, Esslingen, Germany). Prior to the conditioning steps, groups 2–13 were aged in a thermocycling machine (Haake W15, Thermo, Willytec, Gräfelfing, Germany) for 5000 cycles between 5 °C and 55 °C (dwell time: 20 s in each bath; transfer time: 10 s; duration of each cycle: 50 s), as described by Wiegand et al. [26].

### 2.2. Surface Conditioning

After thermal cycling, groups 2–5 were roughened with a 40 µm diamond bur (4325 L, Intensiv, Grancia, Switzerland). Standardized grinding under water cooling with 0.1 mm removal of the surface was guaranteed by attaching the hand piece (40,000 rpm) holding the diamond bur to an apparatus, which maintained load (100 g) and vertical position during preparation [26,34]. Groups 6–9 were sandblasted with 50 µm aluminum oxide (Al_2_O_3_) particles (Airflow Handy 2+, EMS, Nyon, Switzerland), and groups 10–13 were sandblasted with silica-coated aluminum oxide (CoJet, 3M ESPE, Seefeld, Germany). Sandblasting was performed for 10 s perpendicularly and at a distance of 5 mm to the composite surface at 2.5 bar air pressure. Loose particles were air blown.

After the mechanical pretreatments described above, groups 2, 6, and 10 were conditioned with the universal adhesive Scotchbond Universal (3M) for 20 s, air dried for 5 s, and light-cured for 10 s (Elipar Deepcure-S, 3M). Groups 3, 7, and 11 were first treated with a silane coupling agent (Monobond Plus, Ivoclar Vivadent, Schaan, Liechtenstein) for 60 s before the universal adhesive was applied as described above. Groups 4, 8, and 12 were treated with Monobond Plus followed by application of a conventional adhesive (Optibond FL Adhesive, Kerr, Orange, CA, USA) for 15 s, air drying for 5 s, and light-curing for 10 s (Elipar Deepcure-S, 3M).

The negative control groups 5, 9, and 13 were only mechanically pretreated by diamond bur abrasion (group 5), aluminum oxide sandblasting (group 9), or silica coating (group 13), without any adhesive conditioning.

### 2.3. Repair Restoration

All groups were restored with three 1.5 mm thick composite increments of Filtek Supreme XTE (3M; color A1E) using cylindrical Teflon molds. Each repair composite increment was leveled with CompoRoller (KerrHawe), and light-cured for 20 s. Prior to microtensile bond strength testing, all specimens underwent a further thermal cycling procedure (5000 times, 5–55 °C) [26,35].

### 2.4. Microtensile Bond Strength Test

Specimens were first cut longitudinally in two directions with a precision cutting machine (Accutom-50, Struers, Denmark) to obtain nine rectangular sticks of approximately 1 mm × 1 mm × 9 mm from the central portion of each specimen, resulting in 54 sticks per group. The exact dimensions of the sticks were measured using a digital caliper (Kisling, Zurich, Switzerland) to calculate the bonding area. After fixation of the sticks at both sides to sandblasted microtensile bond strength jigs with superglue (Renfert, Hilzingen, Germany), microtensile bond strength testing was determined with a universal testing machine (Z010, Zwick, Ulm, Germany). A constant tension load was applied at a crosshead speed of 1 mm/min, and load at fracture was recorded. Calculation of the microtensile bond strength (MPa) was performed by dividing the load at failure (N) by the bonding area (mm^2^).

### 2.5. Failure Type Analysis

Failure type analysis was performed with a stereomicroscope (M3B, Wild, Heerbrugg, Switzerland) at 25× magnification. Failure types were categorized as cohesive (within the substrate or within the repair composite), adhesive (between the substrate and repair composite), or mixed failure (involving both the interface and composite material).

### 2.6. Statistical Analysis

The microtensile bond strength of specimens which failed prior to testing (pre-test failures) was set at 0 MPa [36]. After checking the assumptions of the parametric approach (normality and homogeneity of variance) using residual plots, data of all groups, except the positive control group, were analyzed by two-way ANOVA to test whether the explanatory variables mechanical pretreatment and adhesive conditioning, as well as their interaction, have a statistically significant influence on repair bond strength. Subsequently, a one-way ANOVA was calculated across all groups. The positive control group was compared with all test groups by post-hoc tests with p-values corrected according to Holm. One-way ANOVA was also used to compare the groups within each mechanical pretreatment, as well as to compare the groups within each adhesive procedure. In the pairwise comparisons, p-values were again adjusted according to Holm. The significance level was set at α = 0.05. All analyses and plots were done with the open-source statistical software R [37].

## 3. Results

**Figure 2:**
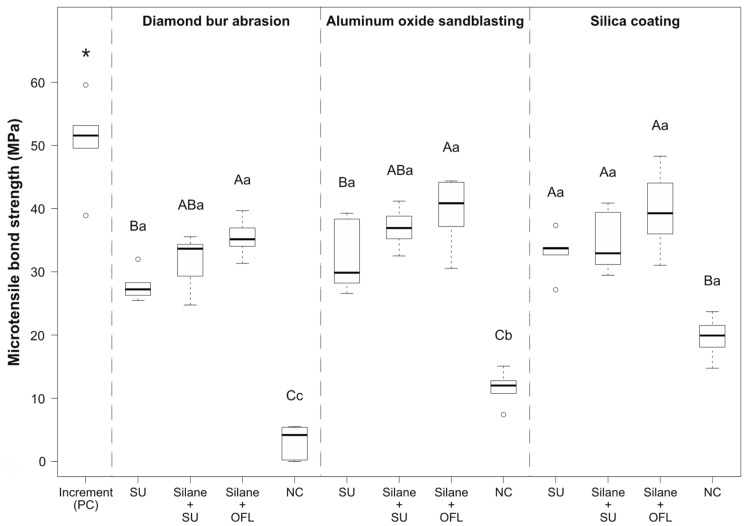
Microtensile repair bond strength (MPa) after mechanical and/or adhesive composite pretreatment. Boxplots show the medians (black lines) with 25 and 75% quantiles (boxes); the whiskers represent 1.5 × IQR (interquartile range), or minima and maxima of the distribution if below 1.5 × IQR; outliers are depicted as circles. Significant differences in repair bond strength between experimental groups within the same mechanical surface pretreatment are indicated with different capital letters, while significant differences within the same adhesive procedure are indicated with different small letters. The asterisk (*) indicates the significantly highest bond strength (positive control group). PC: positive control (incremental bond strength); SU: Scotchbond Universal; OFL: Optibond FL; NC: negative control.

**Figure 3:**
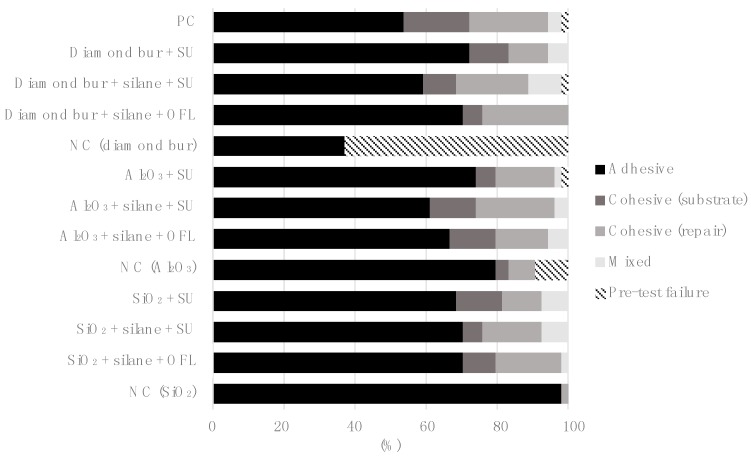
Distribution of failure modes per group, given in percentage (%). Adhesive failures were the predominant failure mode of all groups (pre-test failures not included). Pre-test failures occurred mainly in the negative control group pretreated with diamond bur. PC: positive control (incremental bond strength); SU: Scotchbond Universal; OFL: Optibond FL; NC: negative control; Al_2_O_3_: Aluminum oxide sandblasting; SiO_2_: silica coating.

The microtensile repair bond strengths of all tested groups are presented in Figure 2. Two-way ANOVA revealed that both the mechanical (*p* < 0.001) and adhesive pretreatment (*p* < 0.001) had a significant influence on the repair bond strength. In addition, a significant interaction between the two factors was observed (*p* = 0.002). The positive control group (group 1, incremental bond strength) achieved the significantly highest bond strength values (50.7 ± 6.8 MPa), while the negative control groups (groups 5, 9, 13) obtained the significantly lowest repair bond strength. After diamond bur abrasion or sandblasting with aluminum oxide, the conventional adhesive in combination with a silane coupling agent reached significantly higher bond strength values than the universal adhesive applied without prior silanization (*p* < 0.001 and *p* = 0.016, respectively). However, after separate silanization, no significant differences in repair bond strength were revealed between the universal adhesive and the conventional adhesive. Repair bond strength of the universal adhesive after prior silanization was not significantly different from that without separate silanization, irrespective of the mechanical surface pretreatment. Furthermore, after silica coating, no significant differences in repair bond strength were observed between the different adhesive pretreatments. No significant differences were observed between the mechanical pretreatments, irrespective of the adhesive conditioning. However, when comparing the negative control groups (no adhesive conditioning), silica coating obtained significantly higher bond strength than sandblasting with aluminum oxide (*p* < 0.001), which, in turn, achieved significantly higher values than diamond bur abrasion (*p* < 0.001). The failure mode distributions of the experimental groups are given in Figure 3 showing that all groups failed mainly at the adhesive interface.

## 4. Discussion

The aim of this study was to investigate the effectiveness of a silane-coupling agent incorporated in universal adhesive on the repair bond strength of aged composite. Many previous investigations revealed that silanization prior to application of an adhesive system can improve the bond strength between the substrate and the repair composite [24,25,26]. Silane coupling agents can establish covalent bonds with exposed inorganic filler particles, and increase wettability of the composite substrate surface allowing enhanced infiltration of the bonding agent used for the repair restoration [25,28]. As a matter of fact, some universal adhesives incorporate a silane coupling agent in their formulation. This approach seems to be promising for simplification of the technique-sensitive composite repair procedure by saving a separate silanization step.

The findings of our study indicate similar effectiveness of a silane-containing universal adhesive used with or without separate silanization on the composite-to-composite repair bond strength. Thus, the first null hypothesis was confirmed. Literature is inconsistent on the effectiveness of silanes integrated in adhesive systems. While some studies revealed no differences in the repair bond strength between universal adhesives used with or without prior silanization [19,38,39], other findings suggest that a separate silanization step prior to the use of silane-containing universal adhesive such as Futurabond U is needed [39]. The here used universal adhesive Scotchbond Universal has a higher pH than Futurabond U [40], which might have promoted better stability of the integrated silane [19,41]. Furthermore, ultra-mild adhesives such as Scotchbond Universal (pH = 2.7) have been shown to be less prone to hydrolytic degradation compared to more acidic systems, leading to favorable bond stability [30,42]. In addition, Scotchbond Universal contains 10-methacryloyloxydecyl dihydrogen phosphate (10-MDP) in its formulation [43]. 10-MDP is an adhesive functional monomer with the potential to bond chemically to metals, zirconia, and tooth tissues by formation of insoluble calcium salts. Chemical bonds between 10-MDP and the zirconia nanofiller particles incorporated in the composite substrate material (Filtek Supreme XTE) might have increased the repair bond strength [44]. 

In the present study, composite substrate surfaces were mechanically pretreated before adhesive conditioning. Previous investigations demonstrated that mechanical pretreatment of the composite substrate surface by diamond bur abrasion or sandblasting results in an increase in surface roughness and enhances the composite-to-composite repair bond strength [16,18,23]. The micromorphology of composite surfaces pretreated with diamond burs or sandblasted with aluminum oxide or silica-coated aluminum oxide has been comprehensively investigated in previous studies using scanning electron microscopy [17,18,45,46], revealing that the mechanical pretreatments established a microretentive surface which enables micromechanical interlocking of the adhesive resin. On the other hand, it has been revealed that omitting mechanical surface roughening prior to adhesive conditioning leads to a considerably poorer bond strength between the substrate and repair composite [16,26].

In groups that were only mechanically pretreated, higher repair bond strength values were recorded for sandblasting with either aluminum oxide or silica-coated aluminum oxide compared to diamond bur abrasion. Consequently, the second null hypothesis was rejected. In accordance with our findings for exclusively mechanical pretreatment, previous studies also demonstrated that aluminum oxide sandblasting and silica coating resulted in higher repair bond strength compared to bur abrasion [17,47]. Sandblasting creates a more irregular and more microretentive composite surface than the use of diamond burs [45]. Furthermore, studies using spectroscopic techniques have shown that silica coating enables the formation of Si–O–Si-bonds between the silica surface and the silane molecule [27,48,49,50], which might additionally increase composite–composite repair bond strength [17]. 

The only mechanically pretreated negative control groups achieved significantly lower repair bond strength than when an adhesive was additionally applied. This observation is consistent with previous studies [19,38,51,52] that affirmed the importance of adhesive pretreatment for composite repairs. Interestingly, when either the universal or conventional adhesive was applied after mechanical pretreatment, no significant differences in repair bond strength were found between sandblasted and bur abraded groups. Thus, the adhesive application could level out differences between mechanical pretreatment methods, as previously reported in the literature [26,53].

In the present study, the well-established conventional adhesive system OptiBond FL (Kerr, Orange, CA, USA) was used as a benchmark adhesive in order to better classify the results of the universal adhesive Scotchbond Universal. This conventional adhesive was chosen because of its proven reliable performance in several laboratory and clinical studies [53,54,55,56]. Our results revealed that after separate silanization, the universal adhesive reached similar bond strength values as the conventional adhesive system. However, if the universal adhesive was applied without a separate silanization step, it only reached similar repair bond strength as the conventional adhesive system when the composite substrate surface was silica coated, but not after diamond bur abrasion or aluminum oxide sandblasting.

The results of the current study show that the predominant failure mode in all experimental groups was adhesive failure, irrespective of the mechanical surface pretreatment, as demonstrated in previous investigations performing microtensile bond strength testing for composite repairs [18,24]. Indeed, microtensile bond strength tests show more adhesive failures compared to other test methods because of a better and more homogeneous stress distribution at the adhesive interface during the test loading, thus exhibiting advantages compared to other conventional tensile bond strength tests [57,58,59]. In comparison to the experimental groups, cohesive failures were more frequently observed in the positive control group (directly adhered composite-to-composite increments), where the bond strength was determined by the formation of an oxygen-inhibited surface layer.

Limitations of the current in vitro study are that only one silane-containing universal adhesive and one composite substrate/repair material were investigated. The results obtained in the present study might therefore not be applicable to other universal adhesives or resin composites with different compositions. Thus, further studies are required to implement standard protocols for simplified composite repair techniques. Despite the above-mentioned limitations of our study, the investigated universal adhesive with integrated silane seems to be a promising candidate for simplified composite repairs, especially in patients with low compliance who require a fast but still reliable restoration.

## 5. Conclusions

Within the limitations of this in vitro study it is concluded that the use of a separate silane coupling agent before application of the investigated universal adhesive does not enhance composite–composite repair bond strength, independent of the mechanical roughening procedure. For composite repairs, the silane-containing universal adhesive can thus be used without a separate silanization step. Furthermore, after sandblasting the composite substrates with silica-coated aluminum oxide, the universal adhesive attained similar repair bond strength values as the conventional adhesive system. The combination of mechanical pretreatment and subsequent adhesive conditioning is crucial for adequate composite repairs.

## Figures and Tables

**Table 1:** Composition of the main materials used in the present study.

Product	Manufacturer	Composition	LOT Number
**Filtek Supreme XTE**	3M, St. Paul, MN, USA	Bis-GMA ^1^, Bis-EMA ^2^, UDMA ^3^, TEGDMA ^4^, PEGDMA ^5^, non-agglomerated/non-aggregated silica (20 nm) and zirconia (4–11 nm) fillers, aggregated zirconia/silica cluster filler (mean cluster particle size: 0.6–10 µm), filler content: 78.5 wt% (63.3 vol%)	N783259/A4D N778961/A1E
**Monobond Plus**	Ivoclar Vivadent, Schaan, Liechtenstein	Alcohol, silane methacrylate, 10-MDP ^6^, phosphoric acid methacrylate, sulphide methacrylate	U18210
**Scotchbond Universal**	3M, St. Paul, MN, USA	Dimethacrylate resins, HEMA ^7^, 10-MDP, Vitrebond Copolymer, filler, ethanol, water, initiators, silane	627522
**Optibond FL Adhesive**	Kerr, Orange, CA, USA	Bis-GMA, GDM ^8^, HEMA, ODMAB ^9^, barium aluminoborosilicate, Na_2_SiF_6_, fumed silicon dioxide	5057827

^1^ Bis-GMA: bisphenol-A-glycidyl-dimethacrylate; ^2^ Bis-EMA: ethoxylated bisphenol-A-glycidyl methacrylate; ^3^ UDMA: urethane dimethacrylate; ^4^ TEGDMA: triethylene glycol dimethacrylate; ^5^ PEGDMA: poly(ethylen glycol) dimethacrylate; ^6^ 10-MDP: 10-methacryloyloxydecyl dihydrogen phosphate; ^7^ HEMA: 2-hydroxylethyl methacrylate; ^8^ GDM: glycerol dimethacrylate; ^9^ ODMAB: 2-(Ethylhexyl)-4-(dimethylamino)benzoate.
